# A vasodilator stress MRI perfusion study: large HDL particle number is independently associated with microvascular function in patients with LDL-C <100mg/dL

**DOI:** 10.1186/1532-429X-15-S1-O26

**Published:** 2013-01-30

**Authors:** Akhil Narang, Nicole M Bhave, Chattanong Yodwut, Giacomo Tarroni, Benjamin H Freed, Emily Estep, Kristen M Turner, Cristiana Corsi, Michael Davidson, Tamar Polonsky, Roberto Lang, Victor Mor-Avi, Amit R Patel

**Affiliations:** 1 Section of Cardiology, University of Chicago, Chicago, IL, USA; 2 Division of Cardiology, Northwestern University, Chicago, IL, USA; 3 University of Bologna, Bologna, Italy

## Background

Abnormalities in total cholesterol (TC), high-density lipoprotein (HDL-C), low-density lipoprotein (LDL-C), and triglycerides (TG) are associated with microvascular dysfunction. Recent studies suggest that lipoprotein sub-fractions better predict atherogenic burden than a routine lipid panel. We sought to determine, whether lipid sub-fractionation is associated with myocardial perfusion reserve index (MPRi), a microvascular function surrogate.

## Methods

Twenty-four adults referred for risk stratification from a lipid clinic (19 males, age 60±14 years) with LDL-C<100mg/dL underwent cardiovascular magnetic resonance (CMR) using a 1.5T scanner (Achieva, Phillips). Short-axis CMR images were obtained at three levels of the left ventricle during first pass of Gadolinium-DTPA (0.075mmol/kg at 4ml/sec) for approximately 50 consecutive heartbeats. Images were acquired using a hybrid gradient echo/echo planar imaging sequence 1 minute after injecting regadenoson 0.4mg and then repeated 15 minutes after injecting aminophylline (125mg) under resting conditions. Time intensity curves generated from stress and rest perfusion images were used to determine the area under the curve (from the start of the upslope to the plateau) for the mid-ventricular slice and the LV cavity. MPRi was defined as stress to rest ratio of mid-ventricular area under the curve, normalized to LV cavity area under the curve. TC, HDL-C, LDL-C, TG, and particle numbers for total HDL, total LDL, small LDL and large HDL were measured using nuclear magnetic resonance testing. The association between MPRi and lipid parameters was examined using univariate linear regression; lipid components statistically correlated with MPRi (p<0.05) were then subjected to multivariate analysis. The associations of HDL-C and large HDL particle number with MPRi were compared in separate multivariable models that each included TG and small LDL particle number.

## Results

Most individuals were receiving statin therapy (88%), 11 (46%) had coronary artery disease, and 11 (46%) had hypertension. Lipid concentrations are listed in the table. Univariable linear regression analysis (see figure) showed that MPRi was significantly associated with HDL-C, TG, large HDL particle numbers and small LDL particle numbers; no significant association was found between MPRi and TC, LDL-C, total LDL particle numbers or total HDL particle numbers (see table). Using multivariate analysis, only large HDL particle number was independently associated with MPRi.

**Figure 1:**
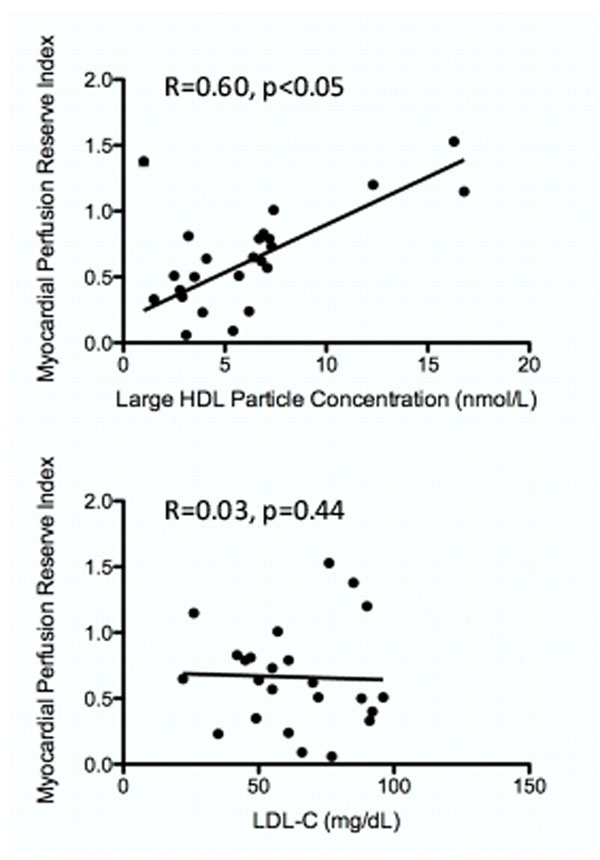
Univariate linear regression between large HDL particle number and myocardial perfusion reserve index (R=0.60, p<0.05) and LDL-C and myocardial perfusion reserve index (R=0.03, p=0.44).

**Table 1:** Lipid concentrations and association between lipid parameters and myocardial perfusion reserve index.

	Lipid concentration	Association with myocardial perfusion reserve index	
***Lipid parameter***	**Mean ± SD**	**R-value**	**p-value**

*Total cholesterol*	144 ± 28 mg/dL	0.02	0.50
*HDL-C*	53 ± 16 mg/dL	0.48	<0.05
*LDL-C*	63 ± 21 mg/dL	0.03	0.44
*Triglycerides*	144 ± 79 mg/dL	0.51	<0.05
*Large HDL particle number*	6.1 ± 4.1 μmol/L	0.60	<0.05
*Total HDL particle number*	35 ± 6 μmol/L	0.19	0.37
*Small LDL particle number*	724 ± 337 nmol/L	0.48	<0.05
*Total LDL particle number*	1004 ± 342 nmol/L	0.35	0.10

## Conclusions

In patients with LDL-C<100mg/dL, only large HDL particle number (and no other lipid component) is independently associated with microvascular function. Further studies are needed to determine if treatments that increase large HDL particle number will improve microvascular function in individuals with well-controlled LDL-C.

## Funding

Astellas Pharma.

